# Characterization of Macrophage-Tropic HIV-1 Infection of Central Nervous System Cells and the Influence of Inflammation

**DOI:** 10.1128/jvi.00957-22

**Published:** 2022-08-17

**Authors:** Blaide M. Woodburn, Krishna Kanchi, Shuntai Zhou, Nicholas Colaianni, Sarah B. Joseph, Ronald Swanstrom

**Affiliations:** a Department of Pharmacology, University of North Carolina at Chapel Hill, Chapel Hill, North Carolina, USA; b Lineberger Comprehensive Cancer Center, University of North Carolina at Chapel Hill, Chapel Hill, North Carolina, USA; c Department of Microbiology and Immunology, University of North Carolina at Chapel Hill, Chapel Hill, North Carolina, USA; d Department of Biology, University of North Carolina at Chapel Hill, Chapel Hill, North Carolina, USA; e Curriculum in Bioinformatics and Computational Biology, University of North Carolina at Chapel Hill, Chapel Hill, North Carolina, USA; f Howard Hughes Medical Institute, University of North Carolina at Chapel Hill, Chapel Hill, North Carolina, USA; g UNC HIV Cure Center, University of North Carolina at Chapel Hill, Chapel Hill, North Carolina, USA; h UNC Center for AIDS Research, University of North Carolina at Chapel Hill, Chapel Hill, North Carolina, USA; i Department of Biochemistry and Biophysics, University of North Carolina at Chapel Hill, Chapel Hill, North Carolina, USA; University of California, Irvine

**Keywords:** HIV-1, HIV-1 CNS inflammation, HIV-1 Env evolution, HIV-1 astrocyte infection, HIV-1 microglia infection, HIV-associated neurocognitive disorders, neuroHIV, macrophage-tropism

## Abstract

HIV-1 infection within the central nervous system (CNS) includes evolution of the virus, damaging inflammatory cascades, and the involvement of multiple cell types; however, our understanding of how Env tropism and inflammation can influence CNS infectivity is incomplete. In this study, we utilize macrophage-tropic and T cell-tropic HIV-1 Env proteins to establish accurate infection profiles for multiple CNS cells under basal and interferon alpha (IFN-α) or lipopolysaccharide (LPS)-induced inflammatory states. We found that macrophage-tropic viruses confer entry advantages in primary myeloid cells, including monocyte-derived macrophage, microglia, and induced pluripotent stem cell (iPSC)-derived microglia. However, neither macrophage-tropic or T cell-tropic HIV-1 Env proteins could mediate infection of astrocytes or neurons, and infection was not potentiated by induction of an inflammatory state in these cells. Additionally, we found that IFN-α and LPS restricted replication in myeloid cells, and IFN-α treatment prior to infection with vesicular stomatitis virus G protein (VSV G) Envs resulted in a conserved antiviral response across all CNS cell types. Further, using RNA sequencing (RNA-seq), we found that only myeloid cells express HIV-1 entry receptor/coreceptor transcripts at a significant level and that these transcripts in select cell types responded only modestly to inflammatory signals. We profiled the transcriptional response of multiple CNS cells to inflammation and found 57 IFN-induced genes that were differentially expressed across all cell types. Taken together, these data focus attention on the cells in the CNS that are truly permissive to HIV-1, further highlight the role of HIV-1 Env evolution in mediating infection in the CNS, and point to limitations in using model cell types versus primary cells to explore features of virus-host interaction.

**IMPORTANCE** The major feature of HIV-1 pathogenesis is the induction of an immunodeficient state in the face of an enhanced state of inflammation. However, for many of those infected, there can be an impact on the central nervous system (CNS) resulting in a wide range of neurocognitive defects. Here, we use a highly sensitive and quantitative assay for viral infectivity to explore primary and model cell types of the brain for their susceptibility to infection using viral entry proteins derived from the CNS. In addition, we examine the ability of an inflammatory state to alter infectivity of these cells. We find that myeloid cells are the only cell types in the CNS that can be infected and that induction of an inflammatory state negatively impacts viral infection across all cell types.

## INTRODUCTION

Neurocognitive impairment is a widespread phenomenon for the 35 million persons living with HIV-1 (PLVH), with 40 to 70% of individuals experiencing some form of HIV-associated neurocognitive disorder (HAND) (1). For untreated PLWH, uncontrolled viral replication in the central nervous system (CNS) drives the most severe forms of HIV-induced neuropathology, resulting in glial dysfunction, death of neurons in multiple regions of the brain, and ultimately, overt dementia (2). Combination antiretroviral therapy (cART) has limited the prevalence of the most severe neuropathology; however, for treated individuals, multiple factors likely contribute to less severe, but still clinically relevant, forms of neuropathology. These factors include chronic activation of resident immune cells, neuronal dysfunction (as opposed to cell death), and stochastic reactivation of virus from latently infected cells (3, 4). Heightened neuroinflammation has been shown to worsen HIV-1 outcomes (5), multiple proinflammatory markers are upregulated in the CNS during infection (6–19), and these changes may alter the environment in ways that impact the susceptibility of CNS cells to infection by HIV-1 within the CNS. Together, these observations demonstrate the complexity of HIV-1 CNS infection and the important role of inflammation.

In the periphery, HIV-1 enters and replicates in cells with a high density of CD4 on their surface, specifically, CD4^+^ T cells. However, in the CNS (largely in the absence of CD4^+^ T cells) the virus can evolve to infect cells with low CD4 densities, specifically resident microglia, perivascular macrophages, and migratory macrophages. This adaptation has been well characterized genotypically and phenotypically and is referred to as macrophage tropism (20–23) due to its ability to more efficiently allow HIV-1 to enter macrophages, which express only a low-density CD4. Perivascular macrophages and microglia are distinct cell types that migrate to the CNS during embryonic development to serve as resident immune cells (24), are permissive to HIV-1 (23, 25–30), and are thought to drive CNS infection locally and significantly contribute to brain pathology (28, 31, 32). These immune cells (perivascular macrophages and microglia) serve similar functions in their respective environments; however, microglia and CNS macrophages occupy separate environments within the brain, interact with different neighboring cells, respond uniquely to inflammatory signals, and may differentially express HIV-1 entry receptors (33). It is not known if macrophage tropism confers the same entry advantage for microglia as it does for macrophages or if the inflammatory environment of the CNS alters the degree to which these cells can be infected. Additionally, it has been shown that inflammation is capable of altering expression of HIV-1 entry receptors (34), but this area of research is underdeveloped, especially as it concerns the effect on macrophage-tropic virus infection.

Astrocytes and neurons play an important, yet indirect, role in pathogenesis during CNS infection. Astrocytes are part of a family of neuromodulating and immunogenic glia cells that support the CNS environment through several functions (35–38). Aberrant astrocyte activation is characteristic of HAND, compromising many of their regulatory functions and potentially contributing to long-term CNS inflammation. There are claims of astrocyte infection by HIV-1 (29, 39–66); however, these glial cells lack the primary CD4 entry receptor (67), making it difficult to conceptualize how the viral and host membranes fuse to allow entry into the cytoplasm for the viral capsid. As for neurons, neuronal degeneration and dysfunction ultimately drive cognitive decline in HAND, with no evidence that this cell type is infected by HIV-1. However, for both astrocytes and neurons, it is not explicitly known if CNS-adapted, macrophage-tropic virus, inflammation, or a combination of the two can mediate susceptibility to HIV-1.

In this study, we characterized HIV-1 infection of cell types found in the CNS in the context of inflammation by infection with pseudotyped viruses representing all three HIV-1 entry phenotypes and with a highly sensitive detection system giving a dynamic range over orders of magnitude. In addition, we compared the infectivity of CNS cells derived from several sources. We found that all myeloid cells were more susceptible to infection by macrophage-tropic viruses than R5 T cell-tropic or X4 viruses and that both interferon alpha (IFN-α) and lipopolysaccharide (LPS) treatment decreased infection due to one or more intracellular restriction mechanisms. We also found that iPSC-derived microglia were less sensitive to IFN-α or LPS-induced viral restriction. Additionally, neither astrocytes nor neurons were susceptible to HIV-1 infection with or without induction of an inflammatory state. We carried out RNA sequencing (RNA-seq) analysis and found that only myeloid cells expressed significant amounts of the HIV-1 receptor and coreceptor, and the level of expression changed only modestly with the addition of IFN-α or LPS. Lastly, transcriptome profiling of CNS cells from different sources revealed that primary cells are, on average, more responsive to inflammatory stimuli. These studies provide important details of virus-host interactions with cells within the CNS that can inform models of viral neuropathogenesis.

## RESULTS

### Validation of R5 and X4 entry phenotypes of Env pseudotyped virus.

**FIG 1:**
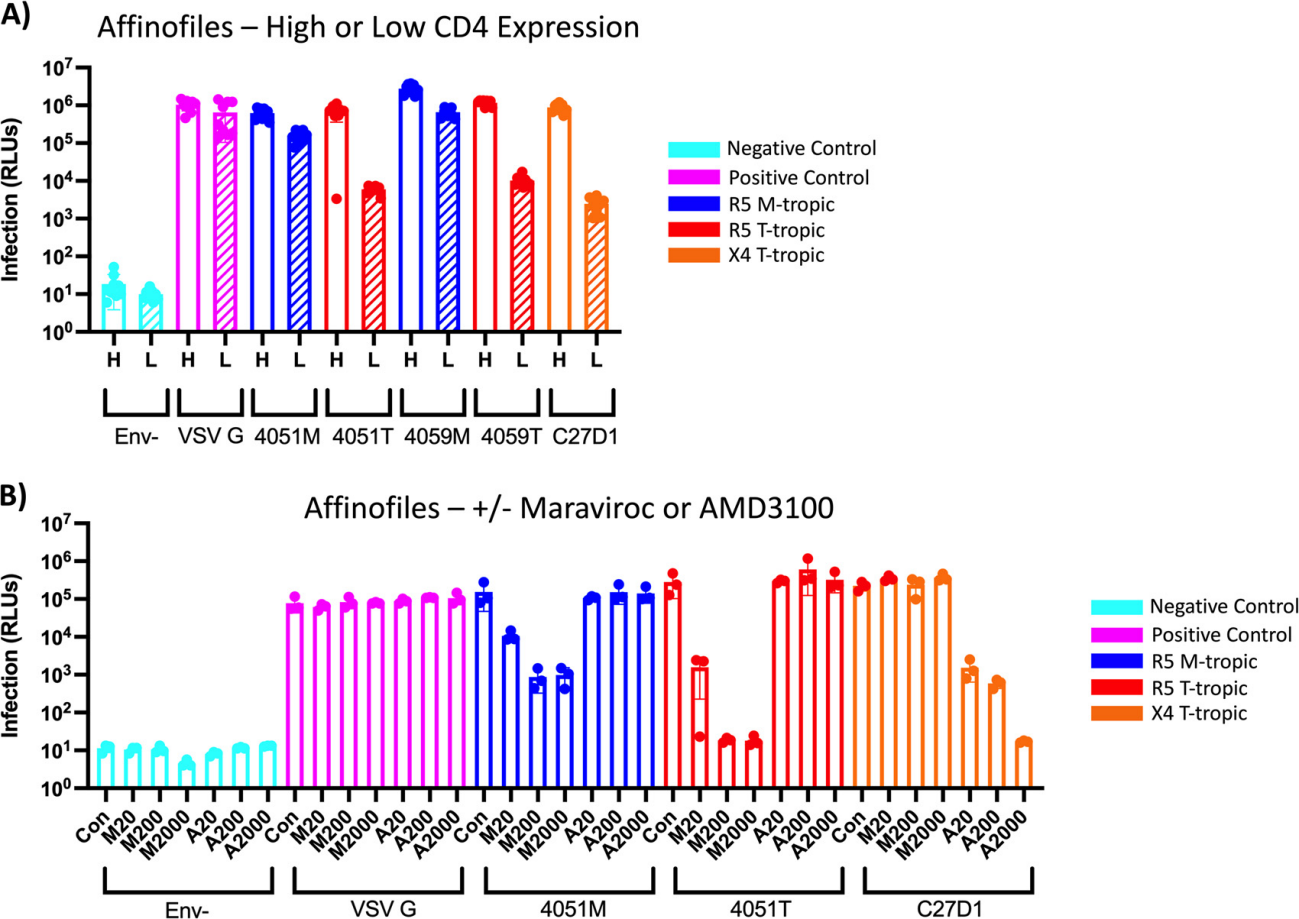
Pseudotyped viruses with Env proteins derived from uncultured virus display CCR5 or CXCR4 coreceptor usage. (A) Affinofile cells induced to express high (H, striped) or low (L, striped) levels of CD4 and high levels of CCR5 were infected with HIV-1 luciferase reporter viruses pseudotyped with patient-derived macrophage-tropic (blue), T cell-tropic (red), or X4 T cell-tropic (orange) envelope proteins. Negative-control viruses (cyan) lacked an envelope protein, whereas the positive-control viruses (pink) were pseudotyped with vesicular stomatitis virus G protein, which is capable of infecting a wide range of mammalian cells. Infection was measured in relative light units (RLUs) via luminometer. (B) Affinofile cells induced to express high levels of CD4 and low levels of CCR5 were treated with 20 nm, 200 nm, or 2000 nm of Maraviroc (M20, M200, M2000) or AMD3100 (A20, A200, A2000) and then infected with HIV-1 luciferase reporter viruses pseudotyped with patient-derived macrophage-tropic (blue), T cell-tropic (red), or X4 T cell-tropic (orange) envelope proteins. Negative-control viruses (cyan) lacked an envelope protein, whereas the positive-control viruses (pink) were pseudotyped with vesicular stomatitis virus G protein, which is capable of infecting a wide range of mammalian cells. Infection was measured in RLUs via luminometer.

In the following studies we used an HIV-1 luciferase reporter virus pseudotyped by cotransfection with viral *env* genes (to express the Env protein) cloned from viral populations *in vivo*. The goal was to study viral entry phenotypes in the absence of any tissue culture adaptation of the virus. We first validated the entry phenotype of the pseudotyped viruses with respect to coreceptor usage and included both a negative-control virus (no surface Env protein/Env–) and a positive control with a virus pseudotyped with the promiscuous entry protein VSV G (vesicular stomatitis virus G protein). We used two pairs of R5 macrophage-tropic (M-tropic) and R5 T cell-tropic (R5 T-tropic) Env proteins for pseudotyping, which originally came from the CSF and blood of participants 4051 and 4059 (23, 68). In addition, we included an X4 T-tropic Env protein pseudotyped virus labeled C27D1 (69); note that this Env entry phenotype is X4 and not dual X4/R5-tropic to allow a clear assessment of the use of CXCR4 as a coreceptor distinct from CCR5 as used by the R5 viruses. We chose these M-tropic clones over the commonly used M-tropic virus BaL because BaL was passaged extensively in culture prior to cloning (70) and has a partially open conformation consistent with tissue culture-adapted viruses (BaL Env pseudoviruses show partial sensitivity to an anti-V3 loop antibody; data not shown); thus, we believe the clones used here are more appropriate for exploring the M-tropic entry pathway in potential target cells. Similarly, the standard X4 virus NL4-3 is also tissue culture adapted (71), making the unpassaged and fully X4-tropic (i.e., not dual) *env* clone C27D1 a more appropriate choice for an X4 entry phenotype as found *in vivo*. In Fig. 1 we validate the entry phenotypes of viruses previously described as being R5 M-tropic (4051M and 4059M), R5 T-tropic (4051T and 4059T), and X4 T-tropic (C27D1).

First, we performed a high-low Affinofile assay to demonstrate the CD4 dependency of the pseduotyped viruses (Fig. 1A). Briefly, in this assay Affinofile cells (72) are induced to express CD4/high+CCR5/high or CD4/low+CCR5/high levels (the cells express CXCR4 constitutively). Infectivity of all viruses is first normalized to infection of cells with a high density of CD4 (CD4/high+CCR5/high). An enhanced ability to enter low-density CD4 cells (CD4/low+CCR5/high) is indicative of an M-tropic entry phenotype, whereas poor infection of cells with a low density of CD4 is indicative of a T cell-tropic entry phenotype. As we have seen previously (23, 73), the CSF-derived HIV-1 Env proteins 4051M and 4059M (previously referred to as 4051C and 4059C) mediate efficient entry of cells expressing a low density of CD4, especially compared to their blood-derived counterparts, 4051T and 4059T (previously referred to as 4051P and 4059P) (Fig. 1A). Similar to the R5 T cell-tropic Env proteins (4051T and 4059T), the X4 T cell-tropic Env protein C27D1 also requires a high density of CD4 for efficient entry and infects poorly at a low density of CD4. As expected, the Env– virus (negative control) showed no significant infectivity and demonstrated the very low background signal achievable in this virus infection system. In contrast, the VSV G pseudotyped virus (positive control) was unaffected by CD4 density as expected.

In Fig. 1B we validated the coreceptor usage of these pseudotyped viruses; we infected CD4/high+CCR5/high Affinofile cells with each of the viruses after first treating the cells for 24 h with 20 nM, 200 nM, or 2,000 nM CCR5 antagonist Maraviroc or CXCR4 antagonist AMD 3100. For all the M-tropic and R5 T cell-tropic pseudotyped viruses, Maraviroc decreased infection up to 100-fold in a dose-dependent manner, whereas AMD3100 did not alter infection, consistent with CCR5 coreceptor usage (data not shown for the 4059 M- and T-tropic pseudotyped viruses). Conversely for the X4 Env pseudotyped virus C27D1, AMD 3100 decreased infection in a dose-dependent manner, whereas Maraviroc had no effect, consistent with exclusive use of the CXCR4 coreceptor. Entry by the VSV G pseudotyped virus was unaffected by these treatments. These data, along with previously published data (69, 73), validate these *in vivo*-derived HIV-1 *env* genes as encoding R5 M-tropic (4051M and 4059M), R5 T cell-tropic, R5 T cell-tropic (4051T and 4059T), or X4 T cell-tropic (C27D1) Env proteins.

### M-tropic HIV-1 Env proteins mediate efficient infection of macrophages, but induction of an inflammatory state with IFN-α or LPS inhibits HIV-1 infection.

**FIG 2:**
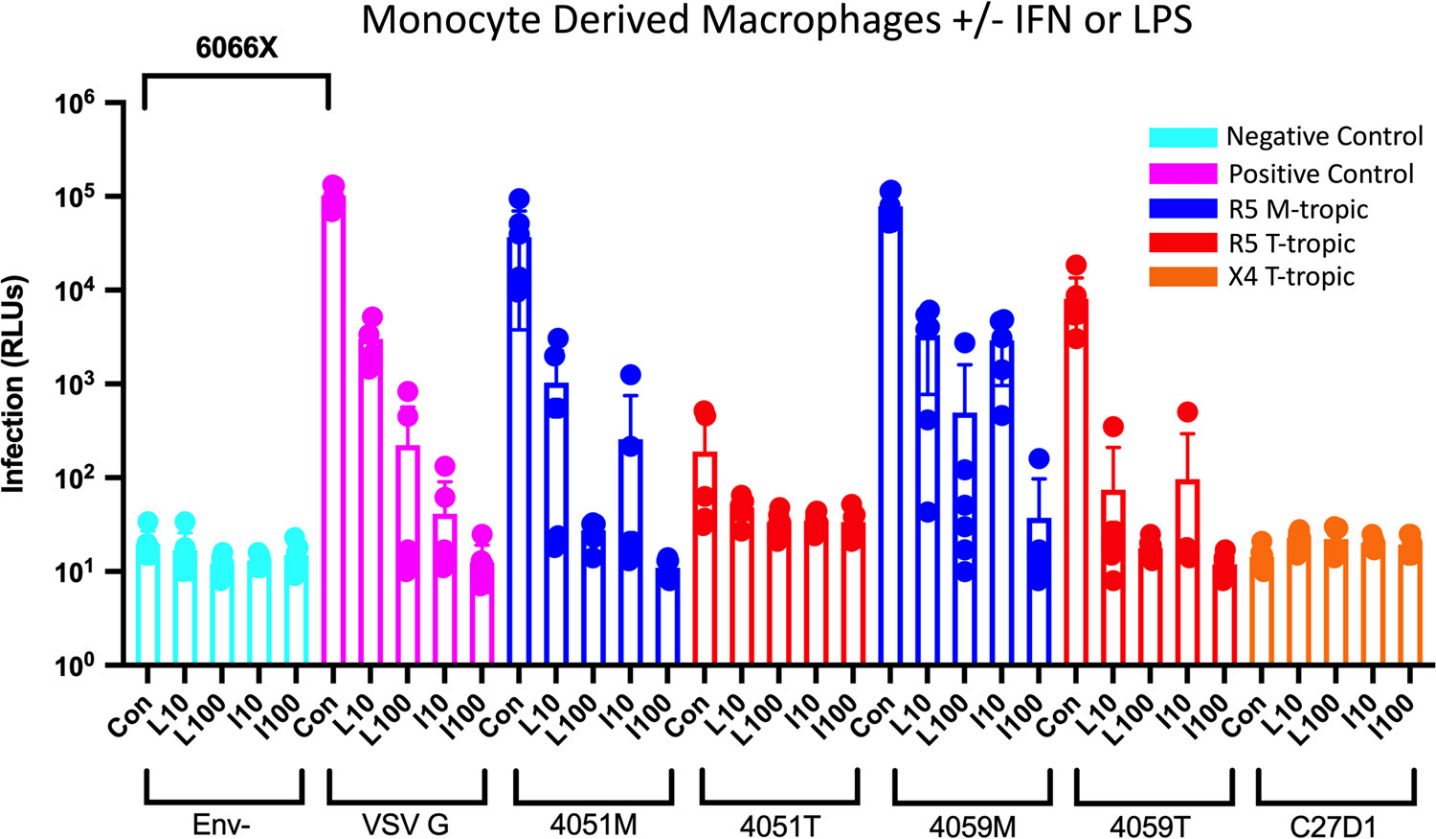
Macrophage-tropic HIV-1 Env proteins confer an entry advantage over R5 T-tropic Env proteins, and LPS and IFN restrict infection in monocyte-derived macrophages (MDMs). MDMs were treated with 10 ng/mL (L10) or 100 ng/mL (L100) lipopolysaccharide (LPS) or interferon alpha (IFN-α) for 24 h and then infected with HIV-1 luciferase reporter viruses pseudotyped with patient-derived R5 macrophage-tropic (blue), R5 T cell-tropic (red), or X4 T cell-tropic (orange) envelope proteins. Untreated control groups are labeled “Con” within each Env group. Negative-control viruses (cyan) lacked an envelope protein, whereas the positive-control viruses (pink) were pseudotyped with vesicular stomatitis virus G protein, which is capable of infecting a wide range of mammalian cells. The average magnitude difference between the positive and negative control was 6,066×. Infection was measured in relative light units (RLUs) via luminometer. *N* = 6 technical replicates.

Macrophages and microglia are permissive to HIV-1 infection and are thought to primarily drive infection within the CNS. We first infected monocyte-derived macrophages (MDMs) with the pseudotyped viruses described above. All virus stocks were normalized to give equivalent levels of infection on Affinofile cells induced to have high levels of surface density CD4 (as measured by relative light units [RLUs]) based on expression of the backbone reporter virus, with the exception of the Env– virus that was used as a negative control. As can be seen in Fig. 2, infection with the Env– virus gave a very low background signal, while infection with the VSV G pseuotyped virus gave a strong signal such that the signal to noise ratio was in the range of 6,000. Both M-tropic viruses (4051M and 4059M) infected at levels similar to that of VSV G, showing that M-tropic viruses have largely overcome the limitation of the low surface density of CD4 for infecting these cells. The 4051 T-tropic Env pseudotyped virus infected poorly at only 10-fold above background and over 100-fold lower than its paired M-tropic Env virus, consistent with having a requirement for a high density of CD4 for efficient entry. Infectivity of the 4059 T-tropic Env pseudotyped virus was approximately 1,000-fold over background but was 10-fold lower than that of the M-tropic Env viruses, indicative of an intermediate phenotype that we have found previously in people with compartmentalized CNS infection within the first 2 years of infection (74) and that can be found in the blood in some people with low CD4 T cell counts (Bednar M, Hauser B, Zhou S, Cohen M, Joseph SB, Swanstrom R, in preparation). Finally, the X4 T-tropic Env pseudotyped virus showed no infectivity over background on MDMs.

HIV-1 infection induces a systemic state of inflammation in both treated and untreated PLWH (2). To mimic such a state, we first treated MDMs with either 10 ng/mL or 100 ng/mL of the Toll-like receptor 4 (TLR-4) ligand lipopolysaccharide (LPS) for 24 h prior to infection; in parallel, we treated other wells of MDMs with either 10 ng/mL or 100 ng/mL of IFN-α. Our initial hypothesis was that induction of an inflammatory state might upregulate CD4 expression and increase infection by R5 T-tropic virus. However, as can be seen in Fig. 2, treatment with either agent had a profound negative effect on infectivity for all viruses. Infectivity of M-tropic Env viruses was reduced by 10-fold or greater at the intermediate level of treatment and at or near background levels at the higher dose of treatment. Treatment with just intermediate concentrations of either IFN-α or LPS lowered infectivity of T-tropic Env viruses to at or near background. Thus, exposure to either IFN-α or LPS induced an inflammatory state that strongly inhibited HIV-1 infection of MDMs. Notably, the same effect was seen with the VSV G pseudotyped virus, suggesting the block to infectivity was intracellular and not at the level of entry, since the VSV G entry protein and the HIV-1 Env entry protein mediate entry through different pathways.

### M-tropic HIV-1 Env proteins mediate efficient infection of different forms of microglia, but induction of an inflammatory state inhibits infection.

**FIG 3:**
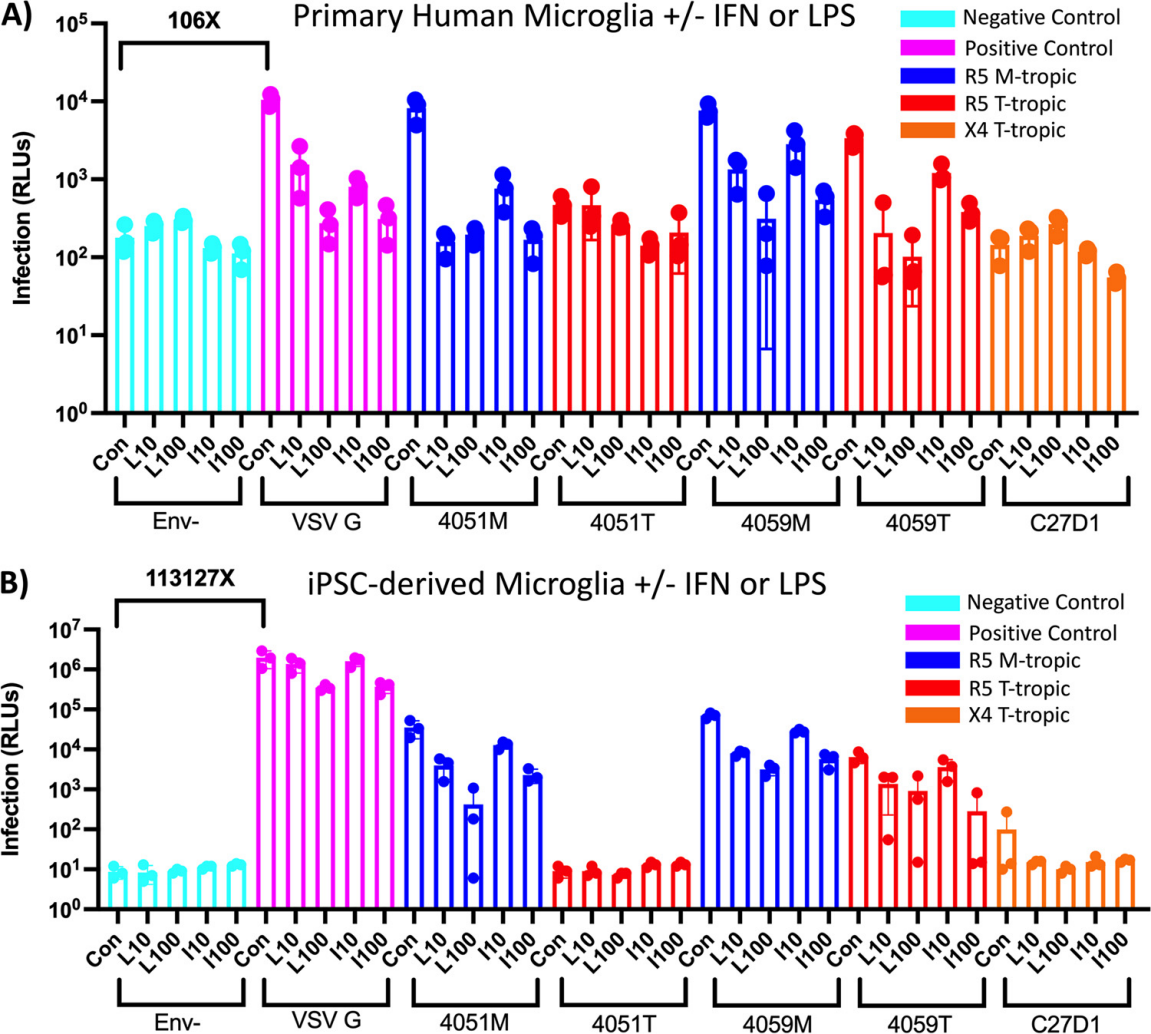
LPS and IFN restrict HIV-1 infection in primary and iPSC-derived microglia, but cell models are not equally permissive. (A and B) Primary human microglia (A) and human iPSC-derived microglia (B) were treated with 10 ng/mL (L10) and 100 ng/mL lipopolysaccharide (LPS) or interferon alpha (IFN-α) for 24 h and then infected with HIV-1 luciferase reporter viruses pseudotyped with patient-derived R5 macrophage-tropic (blue), R5 T cell-tropic (red), or X4 T cell-tropic envelope proteins. Untreated control groups are labeled “Con” within each Env group. Negative-control viruses (cyan) lacked an envelope protein, whereas the positive-control viruses (pink) were pseudotyped with vesicular stomatitis virus G protein, which is capable of infecting a wide range of mammalian cells. The average magnitude difference between the positive and negative control was 106× for primary microglia and 113,127× for iPSC-derived microglia. Infection was measured in relative light units (RLUs) via luminometer. *N* = 3 technical replicates.

Microglia are the resident form of myeloid cell in the CNS and, similar to macrophages, are known to express a low density of CD4 on the cell surface (23, 27, 67, 75), making them a potential target of infection within the CNS. To assess their susceptibility to infection by HIV-1, we infected primary microglia (pMGL) with the same set of viruses and under the same conditions used to infect MDMs. As can be seen in Fig. 3A, overall infection of pMGL was less efficient than infection of MDMs, with the levels of infectivity of the VSV G virus and the M-tropic Env viruses down approximately 10-fold in pMGLs. Our panel of Env pseudotyped viruses displayed the following infection patterns on pMGLs (Fig. 3A) and MDMs (see Fig. 2): (i) M-tropic Env viruses infected at levels similar to that of VSV G virus, (ii) the 4051 T-tropic Env virus infected at low levels close to background, (iii) the 4059 T-tropic virus, with its intermediate phenotype, infected at levels approaching (but not reaching) that of the M-tropic viruses, and (iv) the X4 T-tropic Env virus (C27D1) showed no infectivity. Collectively, these data demonstrate that macrophage tropism confers an entry advantage in microglia similar to what is observed in macrophages. Further, the differential infectivity of M- and T-tropic viruses suggests that microglia express lower densities of CD4 than is optimal for T-tropic viruses.

As noted above, we were interested in testing a model where induction of an inflammatory state would increase infection of cells in the CNS, possibly by inducing expression of the entry receptor CD4 or coreceptors CCR5 or CXCR4. However, similar to MDMs, treatment of pMGL with IFN-α and LPS had a profound negative effect on infectivity (Fig. 3A). We observed dose-dependent decreases in infection across 4051M, 4059M, and 4059T viruses, with the highest concentration of treatment bringing infection signals to or near background. These data suggest that the antiviral effects of IFN-α and LPS observed in MDMs extend to pMGL. Additionally, the VSV G pseudotyped virus showed a dose-dependent decrease in infection in response to both IFN-α and LPS, suggesting an intracellular restriction that spans myeloid cell types. As seen with MDMs, the X4 T-tropic Env virus showed no infectivity, demonstrating that pMGL lack the CD4 densities necessary for efficient infection by this virus.

We next explored an alternative form of microglia-type cells, i.e., microglia induced from pluripotent stem cells (iMGL from iPSC); the iMGL were also infected with the same virus panel after treatment with IFN-α or LPS (Fig. 3B). Infection of iMGL showed similar patterns to MDMs and pMGL with M-tropic Env pseudotyped viruses infecting at higher rates than their T-tropic pairs. 4059T also demonstrated its intermediate M-tropic phenotype on iMGL by infecting at only 10-fold lower than 4059M. Also, the X4 T-tropic pseudotyped virus did not produce a significant signal above background. However, the response of iMGL to IFN-α and LPS differed from that of MDMs and pMGL, with IFN-α and LPS eliciting fewer significant antiviral responses to all HIV-1 Env and VSV G pseudotyped viruses than was observed in both MDM and pMGL, suggesting that iMGL may be less responsive to inducers of inflammation compared to pMGL.

### Astrocytes are not infected by HIV-1 that utilizes M-tropic or T-tropic Env proteins for entry.

**FIG 4:**
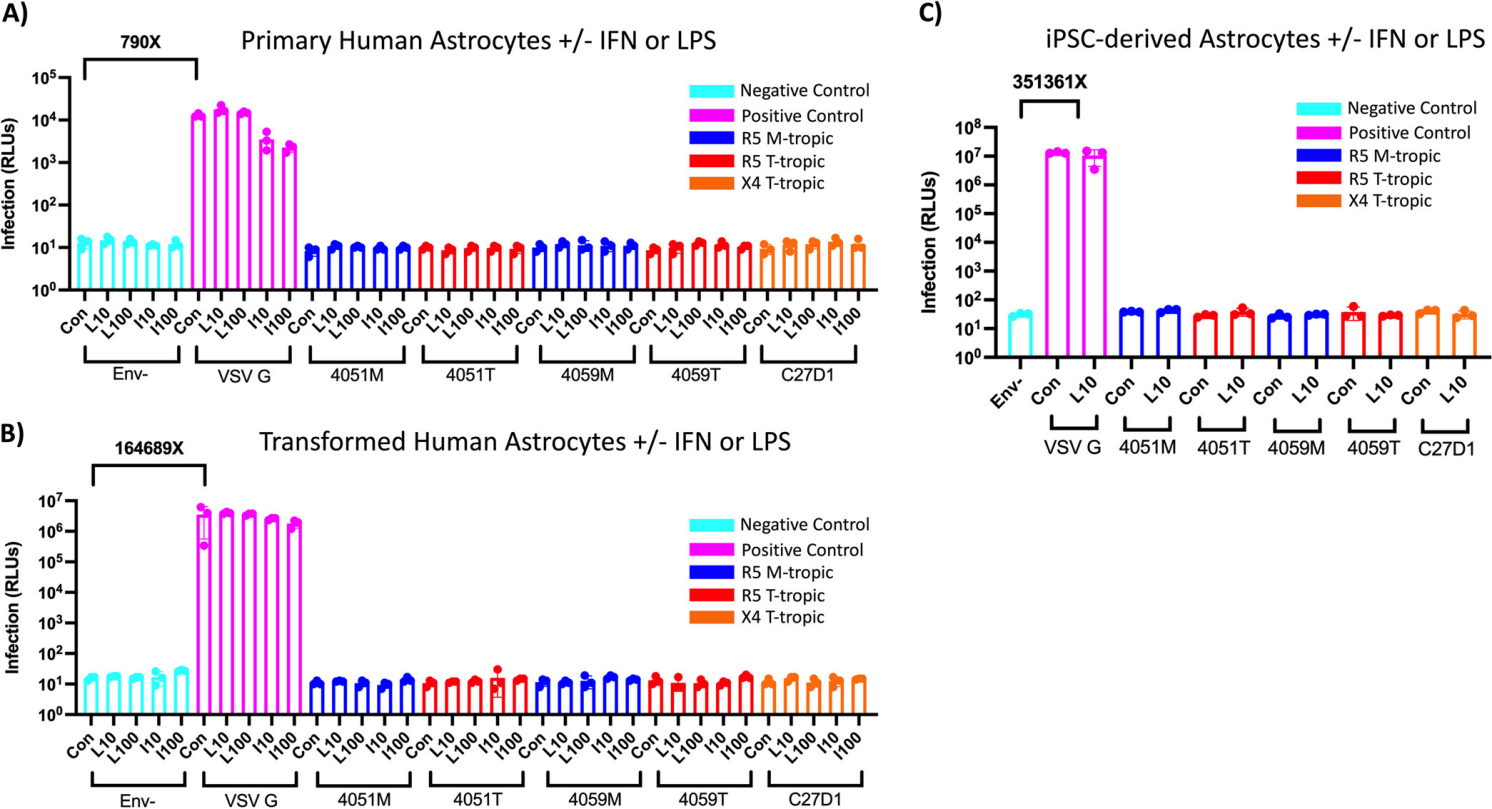
Astrocytes are not infected by macrophage-tropic or R5 or X4 T cell-tropic HIV-1, and inflammation does not potentiate infection. (A to C) Primary astrocytes (A), transformed human astrocytes (B), and human iPSC-derived astrocytes (C) were treated with 10 ng/mL and 100 ng/mL lipopolysaccharide (LPS) or interferon alpha (IFN-α) for 24 h and then infected with HIV-1 luciferase reporter viruses pseudotyped with patient-derived R5 macrophage-tropic (blue), R5 T cell-tropic (red), or X4 T cell-tropic envelope proteins. Untreated control groups are labeled “Con” within each Env group. Negative-control viruses (cyan) lacked an envelope protein, whereas the positive-control viruses (pink) were pseudotyped with vesicular stomatitis virus G protein, which is capable of infecting a wide range of mammalian cells. The average magnitude difference between the positive and negative control was 790× in primary astrocytes, 164,689× in transformed astrocytes, and 351,361× for iPSC-derived astrocytes. Infection was measured in relative light units (RLUs) via luminometer. *N* = 3 technical replicates.

Aberrant astrocyte activation is characteristic of HIV-1 infection of the CNS and demonstrates the involvement of this cell type in CNS pathology. However, claims of direct astrocyte infection by HIV-1 (29, 39–66) are difficult to interpret since these cells lack the CD4 entry receptor. To determine the susceptibility of astrocytes to infection by HIV-1 in the context of inflammation, we attempted to infect primary human astrocytes (pASTRO) with M-tropic or T cell-tropic HIV-1 before and after treatment with either IFN-α or LPS (Fig. 4A). Env– negative controls showed a background of approximately 10 RLUs, whereas VSV G pseudotyped viruses successfully infected pASTROs at 10^4^ RLUs, demonstrating the large dynamic range of our assay and the sensitivity to detect rare infection events. Additionally, successful infection using VSV G pseudotyped viruses demonstrates that pASTROs do not have an intrinsic barrier to expressing the HIV-1 genome, as the luciferase-based infection signal is dependent on transcription from the HIV-1 long terminal repeat (LTR). Importantly, we did not observe infection of pASTROs using either M-tropic or T-tropic pseudotyped viruses above the level of our negative control (Env–) viruses. Additionally, the X4 T-tropic virus did not infect pASTROs, suggesting that even viruses that utilize this alternative coreceptor cannot infect these glial cells.

Further, treatment with either IFN-α or LPS did not potentiate infection in pASTROs. However, IFN-α treatment did decrease infection with VSV G pseudotyped virus in pASTROs in a dose-dependent manner, suggesting that IFN-α facilitates a general and intracellular antiviral response in both myeloid cells and astrocytes. LPS treatment did not potentiate infection in pASTROs and, unlike myeloid cells, did not decrease infection of the VSV G pseudotyped virus, suggesting that these cells are not responsive to TLR-4-mediated LPS signaling.

The susceptibility to infection of a transformed astrocyte-like cell line (tASTRO, ABM immortalized human astrocytes; Fig. 4B) and iPSC-derived astrocytes (iASTRO; Fig. 4C) was also explored in this study. Due to a limited number of iASTROs available, a smaller infection panel that included one concentration of 10 ng/mL LPS was used. Similar to pASTROs, infection of iASTROs and tASTROs resulted in a large dynamic range of between our positive and negative controls, with VSV G infection resulting in values of 10^6^ to 10^7^ RLUs with a background of approximately 10 RLUs. Using both M-tropic and T-tropic viruses, we were unable to detect any infection values above our negative control in either cell model. Additionally, these cells were not permissive to the X4 T-tropic virus. Treatment with IFN-α did not potentiate infection with any virus but resulted in decreased infection in the VSV G pseudotyped virus in pASTROs. This effect was more modest in the tASTROs, suggesting these transformed cells may be less responsive to inflammatory inducers (as seen with iMGL). LPS treatment of iASTROs or tASTROs did not potentiate infection across the HIV-1 Env panel and, similar to pASTROs, did not result in reductions in VSV G pseudotyped virus infectivity. Taken together, these results show that multiple models of astrocytes are not infected with either M-tropic or T-tropic viruses and that induction of an IFN-α or LPS-induced inflammatory state does not potentiate infection.

### Neurons are not infected by HIV-1 that utilizes M-tropic or T-tropic Env proteins for entry.

**FIG 5:**
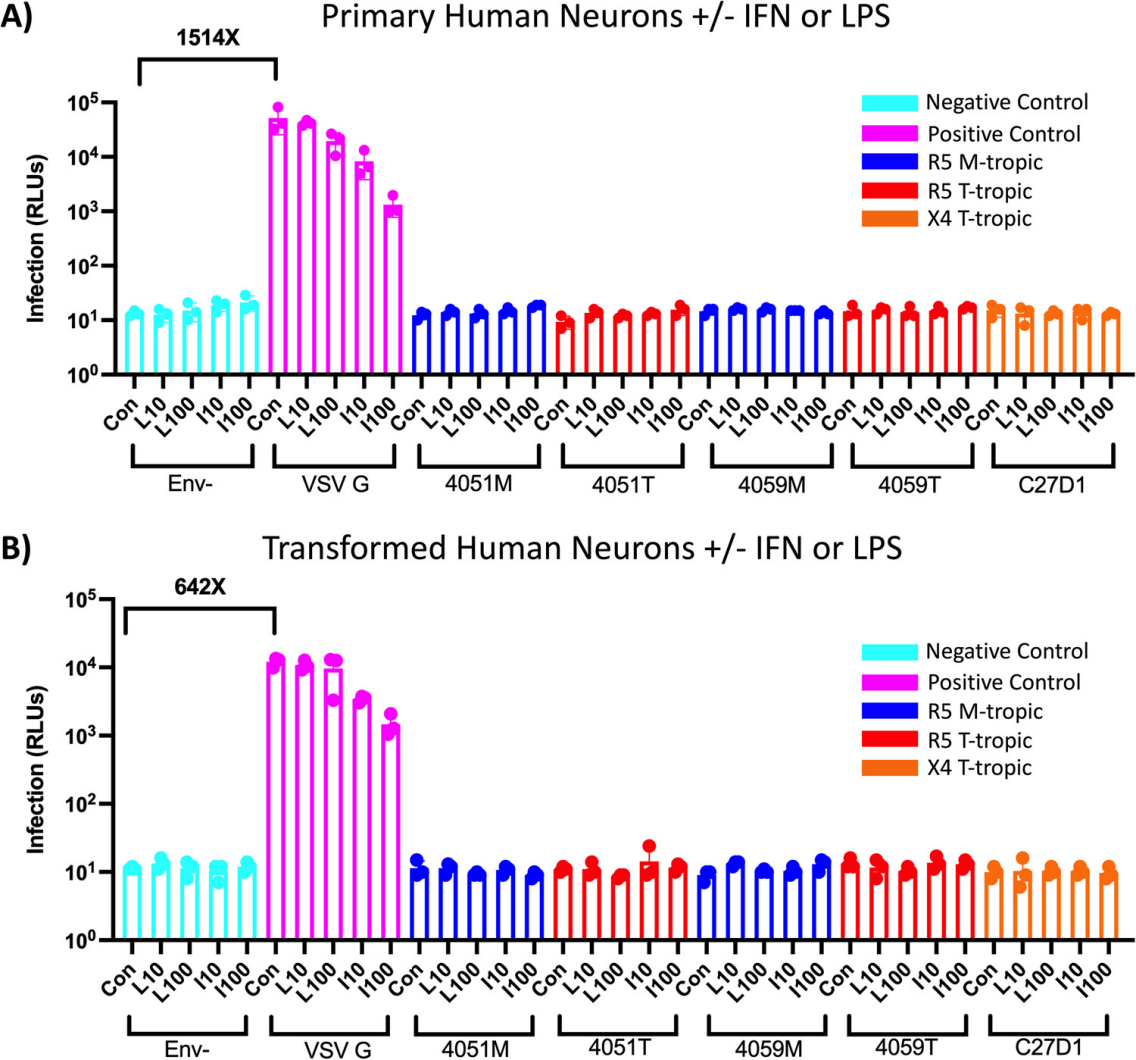
Neurons are not infected by macrophage-tropic or T cell-tropic HIV-1, and inflammation does not potentiate infection. (A and B) Primary human neurons (A) and transformed human neurons (B) were treated with 10 ng/mL and 100 ng/mL lipopolysaccharide (LPS) or interferon alpha (IFN-α) for 24 h and then infected with HIV-1 luciferase reporter viruses pseudotyped with patient-derived R5 macrophage-tropic (blue), R5 T cell-tropic (red), or X4 T cell-tropic envelope proteins. Untreated control groups are labeled “Con” within each Env group. Negative-control viruses (cyan) lacked an envelope protein, whereas the positive-control viruses (pink) were pseudotyped with vesicular stomatitis virus G protein, which is capable of infecting a wide range of mammalian cells. The average magnitude difference between the positive and negative control was 1514× in primary neurons and 642× in transformed neurons. Infection was measured in relative light units (RLUs) via luminometer. *N* = 3 technical replicates.

Given our discordant results regarding infection of astrocytes compared to previous reports (reviewed by Li et al. [54] and Al-Harti et al. [76]) and our interest in testing whether induction of an inflammatory state could potentiate HIV-1 infection, we also used our panel of viruses to infect neurons even though they have been reported not to be permissive to HIV-1 (77). Similar to astrocytes, primary neurons (pNEU) were refractory to infection by HIV-1 Envs, regardless of tropism (Fig. 5A). These cells were infected with the VSV G pseudotyped virus, documenting the ability to express the HIV-1 genome. In addition, IFN-α and LPS treatment did not potentiate infection in pNEU. IFN-α treatment did, however, produce a dose-dependent decrease in VSV G-pseudotyped virus infection, aligning with our previous observations in myeloid cells and astrocytes that an IFN-induced antiviral state is conserved across CNS cell types.

To test an alternative model of neurons, a transformed neuronal cell line (tNEU, SH-SY5Y immortalized neuroblastoma) was subjected to the same virus panel and inflammatory treatments (Fig. 5B). VSV G pseudotyped virus successfully infected these neurons at 10^4^ RLUs, similar to pNEUs. Conversely, tNEUs were not infected with any M-tropic or T-cell-tropic Env-pseudotyped virus, nor did treatment with LPS or IFN-α facilitate infection. IFN-α treatment did result in a dose-dependent decrease in VSV G pseudotyped virus infection, emphasizing a conserved IFN-dependent antiviral restriction across myeloid cells, astrocytes, and neurons.

### Analysis of receptor and coreceptor transcript expression in different cell types and after exposure to inducers of inflammation using RNA-seq.

Uninfected aliquots of the cells described above were used in RNA-seq analysis to look at general expression patterns with and without exposure to inducers of inflammation to determine whether an inflammatory environment might impact expression of the HIV-1 primary receptor CD4 and the coreceptors CCR5 and CXCR4. Expression is one approach to addressing the question of cell phenotype; examining surface protein levels represents a more direct approach but can be difficult given low levels of expression, limited number of primary brain cells, and dealing with the preparation of differentiated cells attached to the culture plate. For these studies we are able to compare changes in levels of receptor/coreceptor transcript expression to the ability of the virus to respond to differences in protein levels as an independent measure of cell phenotype.

**FIG 6:**
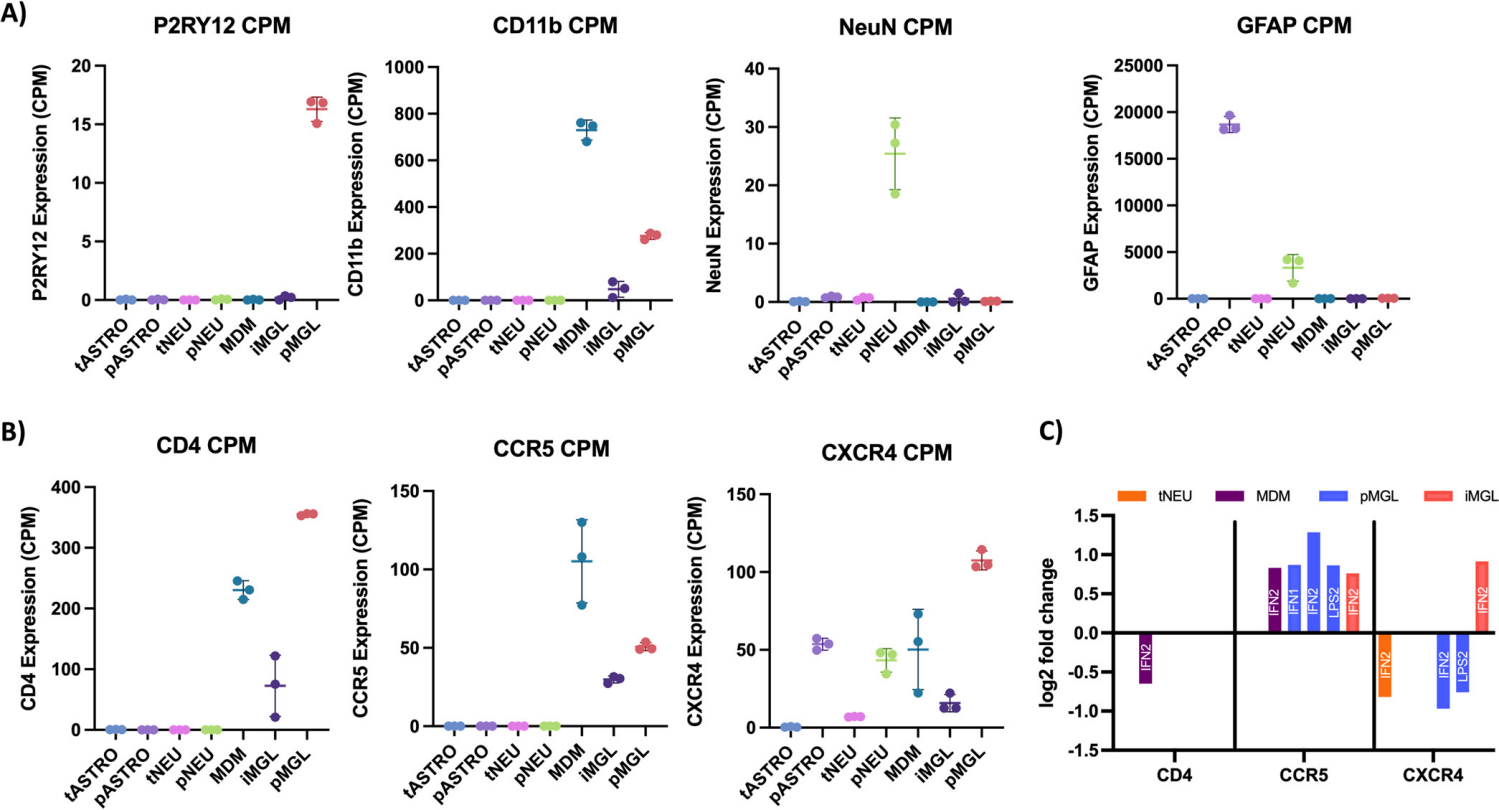
Inflammation induces modest changes in HIV-1 entry receptor transcript expression. (A and B) Following treatment, mRNA sequencing was performed in parallel, noninfected samples to determine basal expression of cell-type-specific markers (A) and HIV entry receptors (B), as well as changes in HIV entry receptor transcripts in response to a 24-h IFN-α or LPS treatment. Counts per million (CPM) were generated in Partek Flow for each gene by normalizing for total reads per samples. (A) The mean CPM (*n* = 3) and standard deviation for each gene are listed for tASTRO (light blue), pASTRO (light purple), tNEU (pink), pNEU (green), MDM (teal), iMGL (dark purple), and pMGL (peach). A) CPM values were generated for P2RY12 (microglia-specific), CD11b (myeloid-specific), NeuN (neuron-specific), and GFAP (astrocyte-specific) to validate the identify of transformed and primary cell types. (B) CPM values were generated for HIV entry receptor CD4 and coreceptors CCR5 and CXCR4. Cell type colors are identical to panel A. (C) Differential expression analysis was performed using DESeq2, and log_2_ fold changes (padj < 0.05) are listed for CD4 (left), CCR5 (middle), and CXCR4 (right) transcripts for tNEU (orange), MDM (purple), pMGL (blue), and iMGL (pink) following LPS or IFN treatment at 10 ng/mL (LPS1/IFN1) or 100 ng/mL (LPS2/IFN2). Transformed astrocytes, primary astrocytes, and primary neurons did not demonstrate changes in HIV-1 entry receptor expression and thus do not appear on this graph.

First, to confirm the identity of our CNS cells, we quantified and normalized specific transcripts within each cell type (Fig. 6A). Expression of the microglia-specific purine receptor (P2RY12) was detected in high quantity in pMGL compared to other cells types, including iMGL. This observation confirms the identity of pMGL, but further calls into question the extent to which iMGL can accurately model pMGL. Further, the myeloid-specific marker CD11b was expressed across all myeloid-derived cells (pMGL, iMGL, and MDMs) but was most highly expressed in MDMs. The neuron marker NeuN was expressed 20 to 30 times higher in pNEUs than other cell types, confirming the neuronal identity of pNEUs. Finally, the astrocyte marker GFAP was expressed much higher in pASTROs than in all other cell types, and higher in pNEUs than the remaining cell types, thus aligning with previous reports that this marker is expressed in neurons but still serves as an accurate marker for astrocytes (78).

Next, basal expression of HIV-1 entry receptors was determined for each CNS cell type (Fig. 6B). As expected, CD4 transcripts were higher across all myeloid cell types, with pMGL expressing the highest levels of the receptor. MDMs showed the second-highest level of expression of CD4, while iMGL expressed the lowest levels of CD4. This suggests that MDMs, on average, express slightly lower levels of CD4 than pMGL. Basal CD4 expression was also quantified in astrocytes and neurons. pASTROs and tASTROs expressed CD4 transcripts at very low levels, as did pNEUs and tNEUs. In combination with our data showing that HIV-1 entry is restricted in astrocytes and neurons, these expression differences demonstrate that astrocytes and neurons do not express levels of CD4 on their cell surface that are sufficient to facilitate biologically meaningful levels of viral entry.

Coreceptor expression was also quantified for each CNS cell type (Fig. 6B). Similar to CD4 expression, CCR5 was expressed across each myeloid cell type. Specifically, MDMs expressed CCR5 transcripts in a range slightly higher than both pMGL and iMGL. Both astrocyte and neuron models expressed little to no CCR5 transcripts. Finally, CXCR4 transcripts were highly variable across CNS cells, with levels ranging from very low to high. pMGL and pASTROs expressed the highest levels of CXCR4 transcripts, while MDM and pNEU expressed moderate levels of CXCR4. tASTROs, tNEUs, and iMGL expressed low to no CXCR4 transcripts. The expression of CXCR4 in myeloid cells at levels only slightly lower than that of CCR5 is notable given that M-tropic viruses use CCR5 for entry and do not appear to evolve from the X4 T-tropic pool of viruses (73).

Although we did not detect infection in astrocyte or neuronal cell lines, MDMs, pMGL, and iMGL were each susceptible to infection, but infection was inhibited with IFN-α and LPS treatment in a dose-dependent manner (Fig. 2 to 5). To determine if viral restriction in myeloid cells could be due to changes in HIV-1 entry receptors, we performed a differential expression analysis using RNA-seq data from IFN-α- or LPS-treated CNS cells. In response to IFN-α or LPS treatment, myeloid cells and tNEUs demonstrated at most small (2-fold or less) changes in HIV-1 entry receptor expression (Fig. 6C). HIV-1 entry receptor expression did not change in response to IFN-α or LPS treatment in tASTROs, pASTROs, and pNEUs.

### Inducers of inflammation significantly alter the cellular transcriptome in primary CNS cells, but these changes are not equally observed in model cell systems.

**FIG 7:**
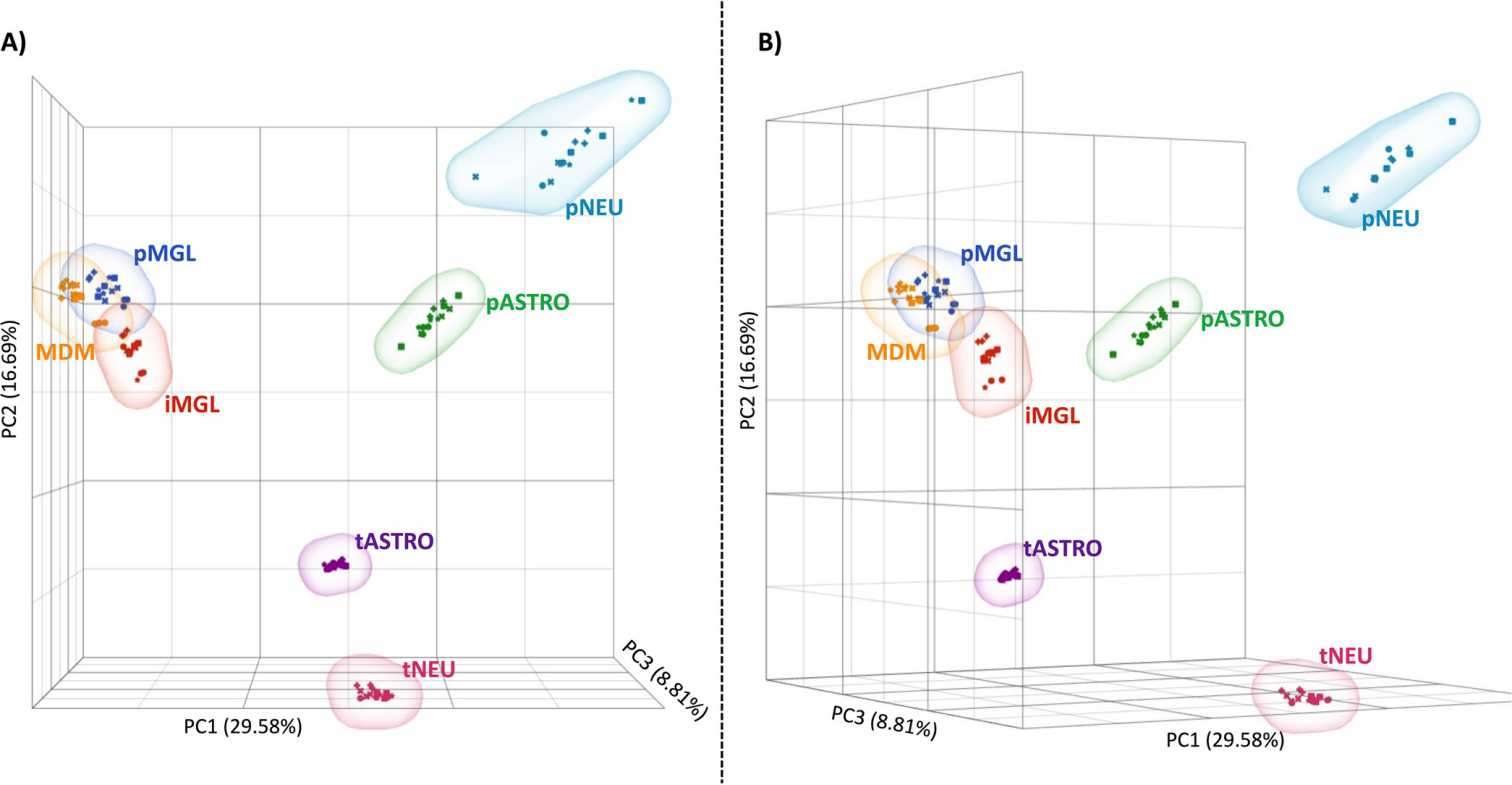
Myeloid-derived cells express similar transcriptomes, but astrocyte and neurons differ significantly by cell type and model. A principal-component analysis (PCA) was generated in Partek Flow using all normalized reads from treated and nontreated pMGL (blue), iMGL (red), MDM (yellow), tASTRO (purple), pASTRO (green), tNEU (pink), and pNEU (teal). Individual replicates for treated (IFN1, squares; IFN2, crosses; LPS1, Xs; LPS2, stars) and nontreated (circles) are displayed for each cell type. Total variance comprised by vector is listed on the correlating axis. (A and B) PC1 versus PC2 is best visualized in a head-on view (A) whereas rotation of the three-vector plot reveals the influence of PC3 (B).

Heightened inflammation is characteristic of acute and chronic HIV-1 infection and for those both on and off antiretroviral treatment. Many studies have described the immunomodulatory role of various CNS cells upon exposure to HIV-1 particles and viral proteins; however, fewer studies have looked across multiple CNS cell models to characterize and compare their transcriptional response to inflammation. To compare the transcriptome of various CNS cell types in their basal state and in the context of inflammation, RNA-seq was performed on uninfected CNS cells following 24-h treatment with IFN-α or LPS. A principal-component analysis (PCA) (Fig. 7A and B) demonstrates that the 55% variation in expression observed across cell types and treatments can be explained by the first 3 components. This PCA was generated by scaling and centering the normalized reads (counts per million [CPM]) and demonstrates that CNS cell types display significant separation based on their transcript expression. PC1, which accounts for the highest variance in the analysis (30%) and mostly clusters samples by cell type, shows that pMGL, MDM, and iMGL cluster closely to the degree of overlapping, demonstrating their transcriptional similarity compared to other cell types. PC1 and PC2 show that both pASTROs and pNEUs are isolated from their corresponding transformed cell models, emphasizing how these transformed cell models are limited in recapitulating the transcriptome of primary cells. This observation is further illustrated by noting that pASTROs and pNEUs cluster closer to each other than to their transformed counterparts. PC3 (visualized in Fig. 7B) further demonstrates the separation between primary and transformed cell lines while also showing that astrocytes (both transformed and primary) are unique compared to neuronal cells. Overall, this analysis reaffirms the similarity of myeloid-derived cells but emphasizes the transcriptional differences between CNS cell types and demonstrates that these transformed cell models do not accurately recapitulate the transcriptional patterns of primary cells. Finally, treatment with either IFN-α or LPS altered transcription but not to the magnitude of the cell-type differences.

**FIG 8:**
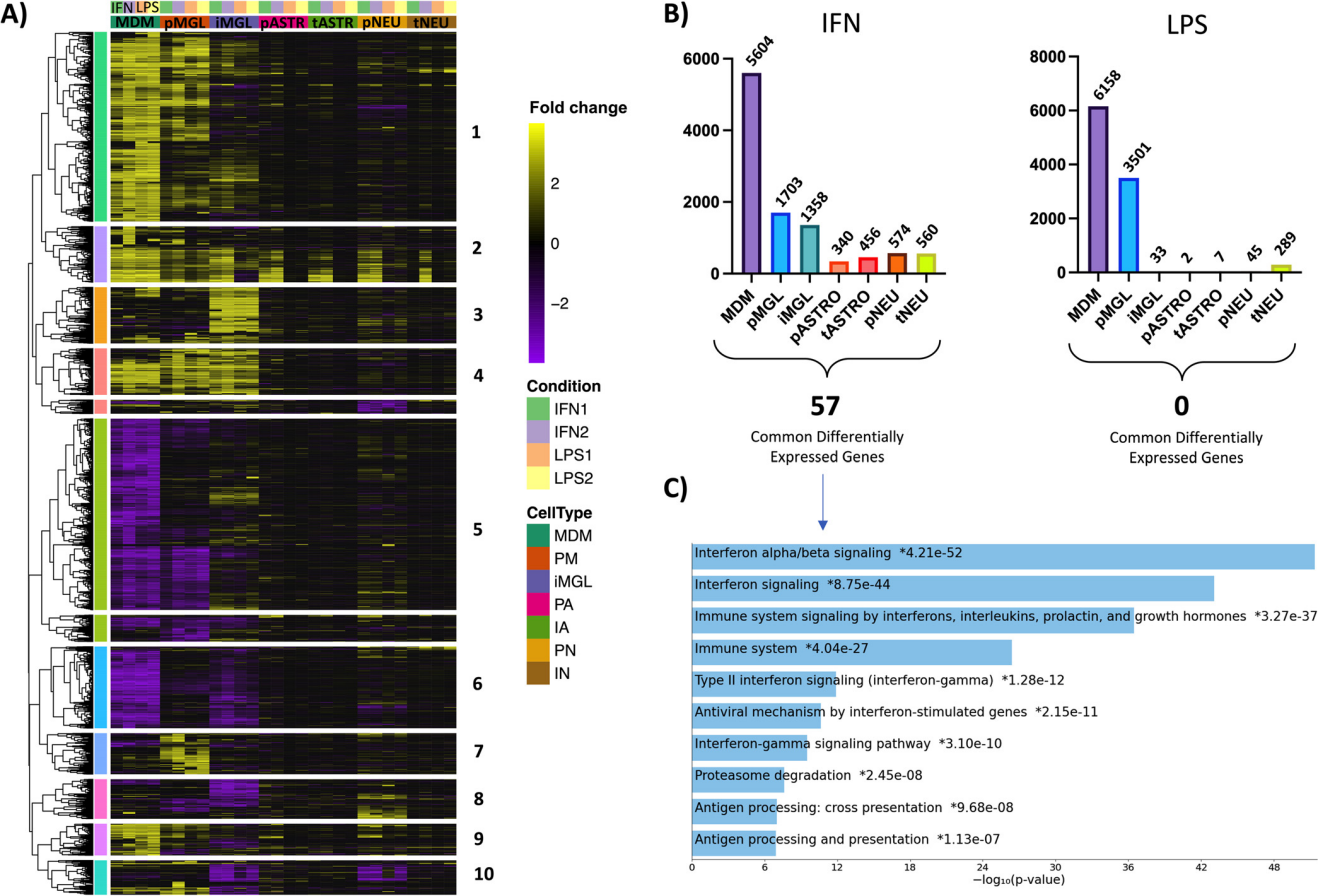
CNS cell types demonstrate nonuniform responses to IFN- and LPS-mediated inflammation. (A) Hierarchical clustering was performed using on the log_2_ fold change of genes across all treatments. Fold changes compared to nontreated samples (upregulation, yellow; downregulation, purple) are listed for each gene across all cell types. Treatment groups include IFN1 (10 ng/mL; green), IFN2 (100 ng/mL; purple), LPS1 (10 ng/mL; orange), and LPS2 (100 ng/mL; yellow). Only genes with a padj of <0.05 and fold change of ±4 in at least one analysis were included. Cluster distances are based on correlation distance with complete linkage. (B) Total differentially expressed genes (padj < 0.05, fold change of ±2) in response to IFN2 and LPS2 are plotted for each cell type. Differentially expressed genes shared between all cell types are listed below the histograms (LPS, 0; IFN, 57). (C) Gene pathway enrichment analysis was performed using Enrichr for the 57 genes commonly differentially expressed across all cell types in response to 100 ng/mL IFN. Log_10_ *P* values representing the strength of correlation are listed for each pathway.

To explore the specific influence of IFN-α and LPS treatment on transcription across all CNS cell types, a hierarchal cluster heatmap was generated using the same differential expression analysis used for Fig. 6C. For each cell type, genes are plotted as the fold change compared to the untreated control for each condition (cutoff at ± 4-fold, adjusted *P* [padj] <0.05 in at least one analysis), where yellow illustrates upregulated genes and purple represents downregulated genes (Fig. 8A). Additionally, the first two columns under each cell type represent IFN1 (10 ng/mL) and IFN2 (100 ng/mL) treatments, and the last two columns represent LPS1 (10 ng/mL) and LPS2 (100 ng/mL) treatments. When making observations across all cell types and treatments, it is convenient to separate the heatmap into clusters, labeled 1 to 10. Broadly, these data demonstrate that myeloid cells are more responsive to IFN- and LPS-induced inflammation than astrocytes or neurons—an observation that aligns with the role of myeloid cells as immunomodulators in the CNS (79). Specifically, genes in cluster 2 were upregulated across all cell types in response to IFN-α, but not LPS. This cluster comprises 226 genes that are most closely associated with IFN-α/β signaling (enrichment analysis not shown) and is consistent with the infection data showing IFN-mediated viral restriction across the CNS cell types.

MDMs had the highest number of differentially expressed genes across conditions (Fig. 8B). MDMs mostly share expression trends with pMGL; however, iMGL and pMGL have expression patterns that differ across multiple clusters (e.g., clusters 1, 3, 5, 6, 7, 8, and 10), for example, cluster 5, which consists of 880 genes that are mostly associated with transmembrane transport of small molecules (gene enrichment not shown). Under inflammatory conditions, genes in this cluster are significantly downregulated in MDMs and pMGL, but are nonresponsive or upregulated in iMGL. This demonstrates that iMGL transcriptional responses to inflammation differ from that of pMGL and provides some hypotheses on the why there are differences in inflammation-mediated viral restriction between primary myeloid cells and iMGL.

To provide a more detailed perspective of the trends observed in our clustering analysis, we quantified the number of differentially expressed genes across cell types for IFN-α and LPS treatments (Fig. 8B). IFN-α treatment resulted in differential expression of 5,604 genes in MDMs and 1,703 genes in pMGL. iMGL differentially expressed slightly fewer genes than pMGL, at 1,358. This trend was emphasized, however, in the LPS treatment groups, where 6,158 and 3,501 genes were differentially expressed in MDMs and pMGL, respectively, but only 33 genes were responsive to treatment in iMGL (Fig. 8B). This was particularly interesting given that MDMs and iMGL expressed similar TLR-4 transcript levels, suggesting either differences in surface expression or discordance in integrating intracellular inflammatory responses. iMGL are increasingly being used as a model for HIV-1 infection of the CNS (80, 81), but their attenuated ability to respond to an inflammatory signal compared to primary cells points to limitations in assuming equivalence between the primary cell and their iPSC-derived counterparts. We have not attempted to test different sources or induction pathways for making iPSC-derived microglia (their general transcription pattern was similar to that of primary microglia, Fig. 7), but our results point to the importance of validating specific pathways under study.

Astrocytes and neurons both displayed moderate responses in their transcriptomes to IFN-α treatment compared to myeloid cells, with 340 and 574 differentially expressed genes, respectively (Fig. 8B). This demonstrates that although astrocytes and neurons are not immune cells, both cell types retain a moderate IFN-α response. In contrast, treatment with LPS resulted in 2 and 45 differentially expressed genes, respectively (Fig. 8B), suggesting that these cells were largely not responsive to TLR-4-mediated LPS stimulation and aligning with overall low TLR-4 transcript expression (data not shown). The exception was LPS treatment of tNEUs, which generated 289 differentially expressed genes, highlighting an unexpected example in which the transformed cell model was more responsive to inflammation than primary cells.

**FIG 9:**
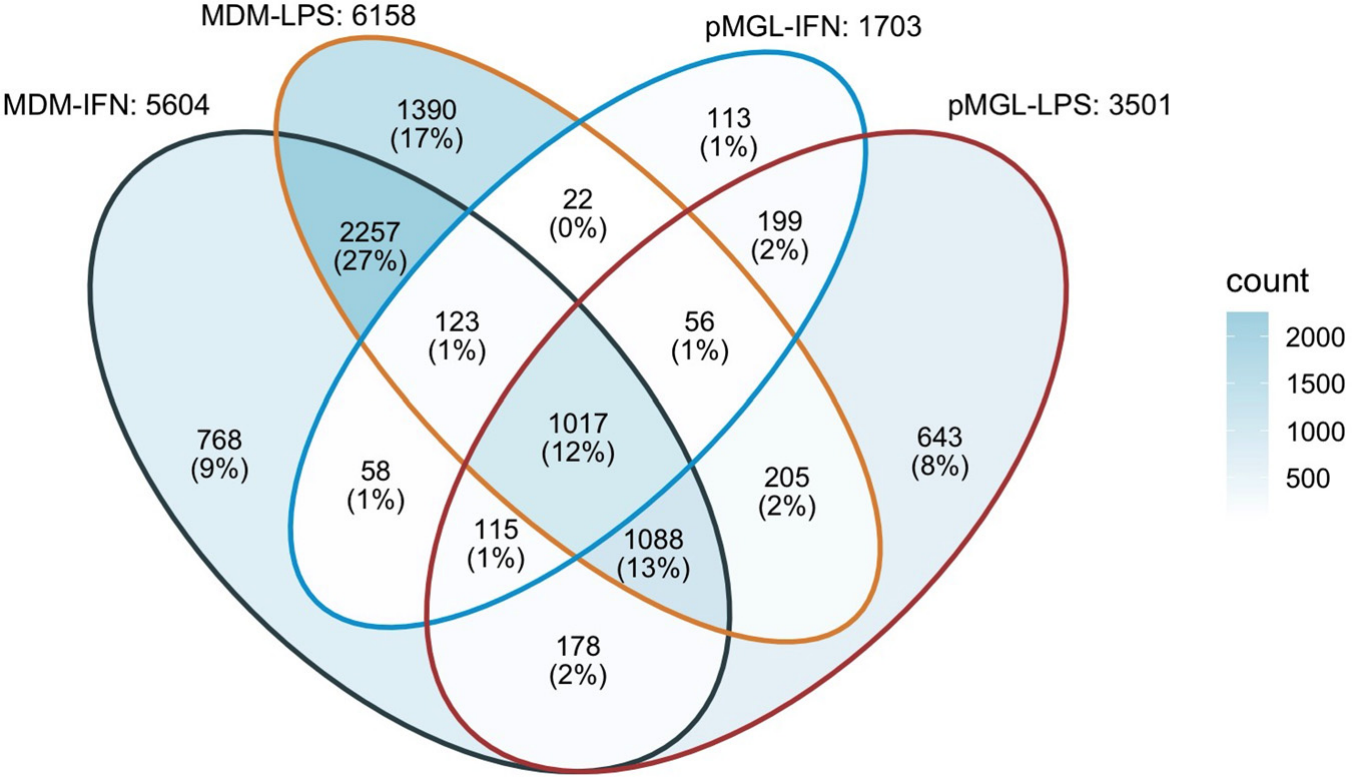
MDMs and pMGL share 1,017 differentially expressed genes across IFN-α and LPS treatments. A Venn diagram was generated to compare differentially expressed genes across all MDM and pMGL treatment groups. The number in each section represents the number of differentially expressed genes shared by the overlapping cell types/conditions, while the percentage represents the group as a fraction of total differentially expressed genes across the analysis.

To investigate if CNS cells expressed conserved responses to IFN-α or LPS inflammation, we examined whether there were transcripts that were differentially expressed across all cell types (Fig. 8B; padj < 0.05, fold change > ±2). There were no genes that were responsive to LPS across all cell types. In contrast, we found 57 genes that were differentially expressed in all cell types when treated with IFN-α. Pathway enrichment analysis of these 57 genes suggests that they function in IFN-α/β signaling, among other immunomodulatory pathways (Fig. 8C). The shared transcripts included known mediators of viral restriction such as *MX1-2*, *OAS1-3*, *IFIT1-3*, and *BST2*, suggesting a conserved IFN-induced antiviral response across CNS cell types. Finally, there were no genes that were differentially expressed across all IFN-treated and LPS-treated groups across all types; however, comparing only primary myeloid cells (e.g., MDM and pMGL) reveals similarity in how these cells respond to inflammation. In Fig. 9 a Venn diagram compares differentially expressed genes across MDMs and pMGL. The number within each section represents the number of differentially expressed genes shared by the overlapping cell type/condition, while the percentage represents the group as a fraction of total differentially expressed genes across the analysis. We found that MDMs and pMGL cells shared 1,313 differentially expressed genes with IFN-α treatment and 2,366 differentially expressed genes with LPS treatment. Interestingly, there were 1,017 genes found to be differentially expressed in both cell types after either IFN-α or LPS exposure, including the 57 shared across all cell types in response to IFN-α. Taken together, these data demonstrate the strong overlap in the response to these two inducers of inflammation between myeloid cell types but present an opportunity to investigate the full range of genes that are regulating the anti-HIV-1 response observed in the infection of these two important myeloid cell types.

## DISCUSSION

Pathogenesis within the CNS during HIV-1 infection is likely multifaceted. There are elements of pathogenesis that occur in the absence of therapy, and some of these features carry over into people on suppressive antiretroviral therapy. In this study, we have carefully examined the susceptibility of CNS cells to HIV-1 infection. We used reporter viruses pseudotyped with HIV-1 Env proteins derived from *env* genes cloned *in vivo* to avoid artifacts of virus adaptation in tissue culture. This system provided us with an assay with a wide dynamic range that permits the quantification of rare or inefficient infectious events. In addition, the use of the promiscuous VSV G entry protein as a positive control allowed us to validate the infectibility of all cell types and distinguish between entry and intracellular blocks to viral replication.

Using these tools, we found both MDMs and microglia to be infectible with enhanced infectivity using M-tropic HIV-1 Env proteins. However, two other CNS-derived cell types, astrocytes and neurons, were completely refractory to infection using HIV-1 Env proteins. Using either transformed cell lines or iPSC-derived cell lines as surrogates for primary cells revealed distinct and important differences with regard to features of HIV-1 infection relative to primary cells. Finally, induction of an inflammatory state had at most modest effects on receptor/coreceptor expression but did induce an intracellular environment that restricted infection up to 100-fold. Taken together, this work gives a comprehensive view of HIV-1 infectivity of CNS-derived cells and the potential role inflammation may play in infection.

### Viruses with M-tropic HIV-1 Env proteins enter macrophages and microglia more efficiently than viruses with R5 T-tropic HIV-1 Env proteins.

Perivascular macrophages and microglia are the most commonly infected CNS resident cells, express low densities of CD4, and are exposed to an inflammatory environment at all stages of treated, and untreated, infection (2, 82). Our infection data demonstrate that M-tropic HIV-1 Envs confer an entry advantage over T cell-tropic Envs when infecting MDM and microglia. This phenomenon has been explored previously with both culture-adapted Env proteins such as BaL (73, 83, 84) and patient-derived M-tropic Env proteins (73, 85, 86). Additionally, a few studies have sought to characterize both macrophages and microglia in the context of HIV-1 (87), but none have used patient-derived HIV-1 Envs or explored the role of inflammation on infectivity. In this study, we demonstrate that patient-derived M-tropic HIV-1 Env proteins confer an entry advantage in two human microglia models. This observation emphasizes the ability of M-tropic Env proteins to mediate infection in the CNS by allowing HIV-1 to more efficiently enter low-density CD4 cells such as microglia that are resident to the CNS. These results suggest that M-tropic HIV-1 variants are likely to have an entry advantage in all myeloid cells that are susceptible to HIV-1.

### IFN-α restricts HIV-1 expression across all CNS cell types, while LPS viral restriction is limited to myeloid cells.

An important question is at what stage(s) in the viral life cycle IFN-α and LPS restrict infection. Our use of VSV G pseudotyped viruses served as a positive control for entry in our infectivity assays and allowed us to examine the intracellular environment for viral DNA synthesis, integration, and LTR-driven expression in cell types that are permissive to HIV-1 and those that are refractory to infection. We observed that IFN-α decreased infection both in experiments where susceptible cells (MDMs, pMGL, and iMGL) were exposed to HIV-1 Env pseudotyped viruses and in all cell types with VSV G pseudotyped viruses. This observation aligns with previous reports that microglia, neurons, and astrocytes each have the ability to respond to IFN (88) and that IFN restricts HIV-1 replication (89, 90) but also highlights a postentry antiviral response conserved across CNS cells. Additionally, we found that LPS decreased infection of HIV-1 Env viruses in susceptible myeloid cells but did not restrict viral replication (using HIV-1 Env or VSV G viruses) in any other cell type. These observations align with a previous account of LPS restricting viral replication in MDMs through release of chemokines (91) or type I interferons (92) but does not explain why the LPS-induced viral restriction observed in myeloid cells but is absent in astrocytes and neurons. Together these observations suggest that the ability of CNS cells to mount a conserved response to inflammatory stimuli is highly dependent on the type of stimuli, with responses to some stimuli (e.g., IFN-α) being highly conserved and others (e.g., LPS) observed in a subset of CNS cells.

### M-tropism, IFN-α, or LPS do not potentiate infection in CD4-negative astrocytes and neurons.

Astrocytes play an integral, yet indirect, role in initiating and maintaining pathology during CNS infection (93); however, there are numerous studies that infer direct involvement via astrocyte infection or propose noncanonical entry mechanisms to allow infection. For example, some reports infer astrocyte infection through detection of proviral DNA in astrocytes isolated from postmortem brain samples using DNAscope and *in situ* hybridization (42, 43). However, these claims have been confounded with data demonstrating that astrocytes are capable of engulfing HIV-1-infected macrophages, providing a more probable explanation of proviral DNA within astrocytes isolated via *in situ* hybridization assays than infection of CD4-negative astrocytes (94). Additionally, there are claims for a CD4-independent astrocyte infection pathway via cell-to-cell transfer from infected T cells to astrocytes, where actively budding virus Env protein binds to CXCR4 and facilitates fusion of the immature viral membrane and astrocyte membrane (54). The specific biochemical mechanism for this phenomenon has yet to be determined which is reported to be insensitive to fusion inhibitors and provides challenges both to the mechanism of membrane fusion and to the viral maturation steps that must occur during budding that are required to confer infectivity. Thus, an additional aim of our study was to explore the influence of M tropism and inflammation on canonically nonpermissive cell types such as astrocytes and neurons. However, using a highly sensitive assay with a wide dynamic range to more accurately detect rare events, we demonstrate that patient-derived, CNS-adapted, M-tropic viruses are incapable of infecting astrocytes. Further, we show that neither IFN-α nor LPS-mediated inflammation can potentiate infection in these cell types.

Similarly, we found that neither M-tropic virus nor inflammation was able to potentiate infection in neurons. Although the dysfunction of these cells primarily drives CNS pathology (95), our data align with the bulk of previous studies that demonstrate that neurons are not infected, regardless of Env protein tropism or inflammatory state.

### HIV-1 entry receptor transcripts are differentially expressed across CNS cell types.

There have been multiple studies directed at quantifying the expression on HIV-1 entry receptors on MDMs, microglia, astrocytes, and neurons separately (25, 75, 96). Using RNA-seq on uninfected cells, we found that each myeloid cell type expressed transcripts for CD4 and CCR5, but these transcripts were not expressed in astrocytes or neurons.

Further, we found that CXCR4 transcripts were variably expressed in multiple CNS cell types, namely, pASTROs, pNEUs, tNEUs, and all myeloid cells (MDMs, pMGL, iMGL). Previous reports have demonstrated expression of CXCR4 on astrocytes and neurons (97); however, this observation appears to have little relevance to HIV-1 infection given that these cells lack CD4. CXCR4 transcripts were expressed at levels similar to those of CCR5 in myeloid cells, an observation also supported by flow cytometry data in MDMs (S. Joseph, unpublished data). However, M-tropic HIV-1 *env* genes cloned from clinical samples use CCR5 as the coreceptor, including those found compartmentalized in the CNS (73). This suggests that coreceptor usage of the virus in the CNS is likely determined by coreceptor usage of the virus that initially seeds the CNS and provides no evidence suggesting that these viruses are commonly selected to switch coreceptors or infect cells by a noncanonical pathway requiring efficient CXCR4 usage.

### Inflammation can induce modest changes in HIV-1 entry receptor transcript expression but does not influence infectivity.

HIV-1 infection involves various forms of inflammation, and some reports suggest that inflammation may alter expression of HIV-1 entry receptors, consequently influencing infectivity (92, 98, 99). However, changes in HIV-1 entry receptor expression in response to inflammation lack consistency. For example, data from Franchin et al. suggest that LPS can decrease infection in MDMs by downregulating CCR5; however, Gordon et al. claim restriction via downregulation of CD4 (98, 99). We explored the influence of two modes of inflammation, and we found differential expression of CD4 in MDMs, CCR5 in all myeloid cell types, and CXCR4 in pMGL, iMGL, and tNEU compared to untreated controls, although all differences were under 2-fold. This observation, and the fact that other myeloid cells experienced viral restriction upon IFN-α treatment with no change in CD4 levels suggests that viral restriction observed in myeloid cells is not due to decreases in CD4 levels. Ultimately, these data demonstrate that CD4 transcript expression is not consistently influenced by IFN-α or LPS inflammation and does not drive the shared antiviral response observed across cell types by changing receptor expression in biologically meaningful ways.

CCR5 was upregulated across all myeloid cells in response to IFN-α treatment and after LPS treatment in pMGL, in direct contrast to previous reports that LPS restricts infection via downregulation of CCR5 in MDMs (98). However, the antiviral effect of IFN-α (and LPS in pMGL) masks any augmentation of infection that may have been realized by increases in CCR5 expression. Inflammation-mediated effects on CXCR4 transcript expression were more variable across cell types, with no effect or modest decreases in primary myeloid cells. Ultimately, RNA-seq analysis of CNS cell types demonstrates that inducers of inflammation are capable of modestly altering the transcript expression of HIV-1 entry receptors. However, the viral restriction observed in our assays appears to be linked not to decreases in expression of the receptors but, rather, to the potentiation of postentry restriction.

### Myeloid cell transcriptomes are similar, and transformed cell lines fail to model primary astrocytes or neurons.

Further exploration of RNA-seq data from basal and inflammatory conditions enabled comparisons of the multiple CNS cells and their model surrogates. Our PCA plot revealed that MDMs, pMGL, and iMGL shared similar transcription patterns, which aligns with previous reports that outline the similarity in expression patterns between these cells (100). However, while astrocytes and neurons collectively show significant distance between one another, the transformed cell lines do not cluster with their primary cell counterparts. pASTROs and pNEUs appear more transcriptionally similar to each other than they are to their transformed cell lines. Other studies have found similar results when comparing transformed cellular models to multiple primary cells (101–103). The transformed cell lines used in this study are widely employed, including studies that focus on transcription (104), inflammation (105), and virology (106–108), including HIV-1 (109). Our data demonstrate that these cells, however, do not accurately model the transcriptome of primary cells, and thus their utility may be limited in some settings.

### CNS cells demonstrate a conserved IFN-mediated antiviral response.

To explore the cause of viral restriction observed in our infection assays, we looked for genes differentially expressed across all cell types in response to IFN-α and LPS treatment. We found no genes that were differentially expressed across cell types in response to LPS; however, 57 differentially expressed genes were shared by all CNS cell types following IFN-α treatment. These genes were highly correlated with interferon α/β signaling and include transcripts canonically involved in viral restriction, such as *MX2*, *OAS1-3*, *IFIT1-3*, and *BST-2*. *MX2* has been shown to be a potent HIV-1 restriction factor in MDMs (110). Beyond these 57 differentially expressed genes across cell types after exposure to IFN-α, there was even greater overlap between the primary myeloid cells, with 1,313 genes differentially expressed when comparing just MDMs and pMGL. Similarly, 2,366 genes are shared in differential expression after exposure to LPS for these two cell types, with 1,017 genes shared between these cell types after IFN-α or LPS exposure. While known restriction factors such as MX2 likely play a role, a more systematic approach is needed to determine the range of host cell factors at work in microglia cells in the context of infection during an inflammatory state.

## MATERIALS AND METHODS

### Cell culture.

**(i) Thawing cells.** All cells (except iPSC-derived lines and MDMs) were thawed from cryopreserved stocks. Briefly, cryovials were thawed in a 37°C water bath, and then the 1-mL cell stock was moved to 5 mL of the appropriate room temperature medium. Cells were spun at 300 relative centrifugal force (RCF) for 5 min, supernatant was removed, and the cells were resuspended in their respective media at the appropriate concentration for plating in a 96- or 24-well format.

**(ii) Affinofiles.** Affinofile cells (72) were plated at 1.8 × 10^4^ cells per well and maintained in Dulbecco’s modified Eagle’s medium (DMEM) with 4.5 g/L glucose (Cellgro) supplemented with 10% dialyzed fetal bovine serum (FBS) (12 to 14 kDa; Atlanta Biologicals) and 50 mg/mL blasticidin (Invitrogen). Cells were induced for expression of CD4 and CCR5 as previously described (73).

**(iii) Astrocytes.** Immortalized human astrocytes (ABM, T0281) were plated at 1.5 × 10^3^ cells per well and cultured in PriGrow IV medium (ABM, TM004) supplemented with 10% FBS, 50 U/mL penicillin, 50 μg/mL streptomycin, 10 ng/mL human epidermal growth factor (EGF), and 1% l-glutamine. Primary human astrocytes (ScienCell, 1800) were plated at 1.5 × 10^3^ cells per well and maintained in astrocyte medium (ScienCell, 1801) supplemented with 10% FBS, 50 U/mL penicillin, 50 μg/mL streptomycin, and 5 mL astrocyte growth supplement (ScienCell, 1852).

iPSC astrocytes were graciously provided by the UNC Pluripotent Stem Cell Core and maintained in differentiation medium until infection. iPSCs were maintained in StemFlex medium (Thermo Fisher Scientific) on Matrigel-coated dishes (Corning). Cells were passaged at a 1:6 ratio twice a week with 0.5 mM EDTA dissociation solution and cultured at 37°C and 5% CO_2_. iPSCs were differentiated into astrocytes as described in Battaglia et al. (111). Briefly, iPSCs were differentiated into neuronal progenitor using neural induction medium (NIM; StemCell Technologies) following the instructions from the manufacturer. At day 12, neuronal rosettes were selected using rosette selection reagent (StemCell Technologies), and neuronal progenitors were plated for differentiation into astrocyte precursors by plating 1 × 10^5^ cells/cm^2^ on poly-ornitine and laminin (PLO/LAM)-coated dishes in STEMdiff astrocyte differentiation medium (StemCell Technologies). Cells were maintained under these conditions for 20 days with medium changes every 48 h and passage every week with Accutase (Millipore). Maturation of the astrocyte progenitor was induced with STEMdiff astrocyte maturation medium (StemCell Technologies). Astrocytes were expanded for up to 120 days and passaged once a week on PLO/LAM-coated dishes, with medium changes every other day. Astrocyte maturation was assessed every 3 weeks with antibodies against GFAP and β3 tubulin. Cell cultures were tested routinely for mycoplasma.

**(iv) Monocyte-derived macrophages.** Monocyte-derived macrophages (MDMs) were generated by first isolating monocytes from a healthy donor blood sample. Approximately 200 mL of blood was collected from each donor into EDTA-treated tubes. Buffy coats were purified using Ficoll-Paque Plus (GE Healthcare) following the manufacturer’s directions. Monocytes were isolated using the negative selection EasySep monocyte isolation kit (StemCell, 19359). Monocytes were plated in a 96-well format at 50,000 cells/well and then differentiated into macrophages over 7 days using RPMI 1640 supplemented with 10% human serum, 100 mg/mL penicillin and streptomycin, and 25 ng/mL recombinant human macrophage colony-stimulating factor (M-CSF; Gibco). A full medium change was performed on day 4, and cells were used for downstream experimentation on day 7.

**(v) Primary human microglia and iPSC microglia.** Primary human microglia (ScienCell, 1900) were plated at 4.8 × 10^3^ cells per well and maintained in microglia medium (ScienCell, 1901) supplemented with 10% fetal bovine serum (FBS), 50 U/mL penicillin, 50 μg/mL streptomycin, and 5 mL microglia growth supplement (ScienCell, 1952). iCell iPSC microglia (FujiFilm, R1131) were plated at 1.5 × 10^3^ cells per well and maintained in iCell glial base medium supplemented with iCell microglia supplements A, B, and C.

**(vi) Immortalized and primary neurons.** Immortalized human neurons (ATCC, CRL-2266) were plated at 1.5 × 10^3^ cells per well and maintained in ATCC-formulated Eagle’s minimum essential medium supplemented with 10% FBS and 100 mg/mL of penicillin and streptomycin. Primary human neurons (ScienCell, 1520) were plated at 5.5 × 10^3^ cells per well and maintained in neuron medium (ScienCell, 1521) supplemented with 10% fetal bovine serum (FBS), 50 U/mL penicillin, 50 μg/mL streptomycin, and 5 mL neuron growth supplement (ScienCell, 1562).

**(vii) Treatment of cells with IFN-α or LPS.** Twenty-four hours prior to infection, cells were treated with IFN-α (Millipore Sigma, SRP4595-100UG) or LPS (Thermo Fisher Scientific, 00-4976-03). Cell medium was prepared with 10 ng/mL or 100 ng/mL IFN-α or LPS and then a full medium change was performed. These concentrations were chosen based on a literature review of INF (over 25 studies) of IFN-α and LPS treatments that elicited the most robust response across CNS or myeloid cell types without sacrificing cell viability. We validated cell viability at the highest concentrations using the Cell TiterGlo viability assay (Promega, G9242).

### Generating pseudotyped viruses.

**(i) Transfections.** The *env* genes used to generate pseudotyped viruses have been described previously (73). HIV-1 Env pseudotyped luciferase reporter viruses were generated by cotransfecting 10-cm dishes of 293T cells with 5 μg of an HIV-1 *env* clone and 5 μg of HIV-1 SubC *Renilla* LucR backbone (obtained from the Division of AIDS, NIAID, through the NIH AIDS Research and Reference Reagent Program) using FugeneHD (Promega, E2311). At 5 h posttransfection, the medium was changed, and the cells were incubated at 37°C for an additional 48 h. Viral supernatants were then harvested, filtered through a 0.45-μm filter, and stored in aliquots at –80°C. Virus stocks were not subjected to multiple freeze-thaw cycles.

**(ii) Virus titers and infections.** The titers of viruses were determined on Affinofile cells with maximum CD4/CCR5 expression. A dilution series was used to determine the linear range of the signal and define the volume needed to achieve an infection giving 800,000 relative light units (RLUs) on Affinofile cells for each pseudotyped virus, a value that is near the upper end of the linear range of the assay.

Each cell type was plated in a 96-well format for infection at manufacturer-suggested cell density to ensure even coating of cells across the bottom of the well. At 24 h prior to infection, cells were treated with IFN-α or LPS at 10 ng/mL or 100 ng/mL. Prior to infection, the cells were washed with phosphate-buffered saline (PBS). Infection experiments were performed by adding the appropriate volume of each virus to cells and performing a 2-h infection at 37°C and 2,000 rpm (i.e., spinoculation [112]), followed by incubation for 2 days at 37°C and then lysing of cells using *Renilla* lysis buffer (Promega, E2810) and analysis for infection by measuring luciferase expression using a luminometer.

### RNA sequencing (RNA-seq).

In parallel to infection experiments, noninfected cells plated in 24-well format were harvested for RNA-seq. Following 24-h IFN-α or LPS treatments, total RNA was harvested from 100,000 to 200,000 cells using an RNeasy RNA isolation kit (Qiagen, 74004). RNA was quantified using a NanoDrop spectrophotometer and TapeStation electrophoresis prior to library preparation. cDNA libraries were prepared using the KAPA RNA HyperPrep kit (Roche, 08098115702). Paired-end RNA sequencing was performed on a NovaSeq S4 platform at a read length of 100 bp, resulting in approximately 60 million reads per sample.

Analysis of the sequencing data was performed using tools available via the UNC-CH Longleaf computing cluster. Raw FASTQ files were then aligned to the GRCh38 human genome (GRCh38.d1.vd1fa from the National Cancer Institute’s Genomic Data Commons [GDC]) using STAR 2.7.6a (113). Quantification of gene abundance for each sample was done with Salmon 1.4.0 (114) using the human transcriptome defined by GENCODE (115) (release 22). Quality control evaluation of the data was done using FastQC 0.11.9 as well as the Picard Toolkit 2.22.4 CollectRnaSeqMetrics, flagstat, and maximum read length tools. Gene level counts were compiled, and a gene was removed from subsequent analysis if it contained fewer than 5 reads across all the samples. Principal-component analysis and relative log expression were calculated to check for any outliers. Normalization and differential gene expression were performed using the DESeq2 (116) 1.22.2 Bioconductor (117) package in R 4.0.3. The Benjamini-Hochberg method was used to correct the *P* values for multiple hypothesis testing. Each cell type was analyzed separately using this methodology for the differential expression analysis (Fig. 6C and Fig. 8). For the transcript quantification in Fig. 6 and PCA plot in Fig. 7 combined, normalized (counts per million + 0.0001) expression values were calculated in Partek Flow and used to generate all CPM normalized graphs.

Pathway analysis was performed using Enrichr (118). Briefly, Enrichr implements three approaches to compute enrichment: (i) the Fisher exact test, (ii) an internally developed correction to the Fisher exact test, where enrichment is computed using the Fisher exact test for many random input gene lists in order to compute a mean rank and standard deviation from the expected rank for each term in each gene-set library, and (iii) a combined *P* value computed using the Fisher exact test with the Z-score of the deviation from the expected rank.

### Data availability.

RNA-seq data were deposited in the NIH Sequence Read Archive (SRA) under BioProject PRJNA856252. Raw RNA-seq reads were deposited in FASTQ file format, and information regarding source organism, isolate, age, and tissue are included in the submission. Raw infection values from luciferase reporter assays are available upon request.
